# Burden of Healthcare-Associated Infections on Mortality Among COVID-19 Hospitalized Patients

**DOI:** 10.3390/jcm14238279

**Published:** 2025-11-21

**Authors:** Corina Voinea, Elena Mocanu, Elena Dantes, Sanda Jurja, Ana-Maria Neculai, Aurora Craciun, Sorin Rugina

**Affiliations:** 1 Doctoral School of Medicine, Faculty of Medicine, Ovidius University of Constanta, 1 University Alley, Campus—Corp B, 900470 Constanta, Romania; corina.badescu2012@gmail.com (C.V.); elena.dantes@365.univ-ovidius.ro (E.D.); sanda.jurja@365.univ-ovidius.ro (S.J.); sorinrugina@yahoo.com (S.R.); 2 Public Health Directorate Constanta, 1 Lacramioraei Alley, 900643 Constanta, Romania; 3 Department of Public Health and Management, Faculty of Medicine, Ovidius University of Constanta, 1 University Alley, Campus—Corp B, 900470 Constanta, Romania; 4 Clinical Hospital of Pneumopthisiology Constanta, 40 Santinelei Street, 900002 Constanta, Romania; 5 Department of Pneumology, Faculty of Medicine, Ovidius University of Constanta, 1 University Alley, Campus—Corp B, 900470 Constanta, Romania; 6 Department of Ophthalmology, Faculty of Medicine, Ovidius University of Constanta, 1 University Alley, Campus—Corp B, 900470 Constanta, Romania; 7 County Clinical Emergency Hospital “St. Apostle Andrew” Constanța, 145 Tomis Boulevard, 900591 Constanta, Romania; 8 Department of Biochemistry, Faculty of Medicine, Ovidius University of Constanta, 1 University Alley, Campus—Corp B, 900470 Constanta, Romania; anamneculai89@gmail.com (A.-M.N.); aurora.craciun@365.univ-ovidius.ro (A.C.); 9 Romanian Academy of Medical Sciences, 1 I.C. Brătianu Boulevard, Sector 3, 030171 Bucharest, Romania; 10 Academy of Romanian Scientists, Ilfov Street, No. 3, Sector 3, 050044 Bucharest, Romania

**Keywords:** COVID-19, healthcare-associated infections, *Clostridioides difficile*, mortality, risk factors

## Abstract

Background: Healthcare-associated infections (HAIs) are a significant public health problem, having a decisive impact on the prognosis of patients hospitalized with COVID-19. In Romania, the absence of a uniform reporting system and the lack of epidemiological data comparable to European standards limit the real assessment of their incidence and consequences. Methods: In this context, the present study aimed to conduct an integrated analysis of the clinical, epidemiological, and microbiological factors involved in the mortality of patients with COVID-19 and HAIs in a county located in southeastern Romania. This research was based on a retrospective observational study that included 295 patients with a confirmed diagnosis of COVID-19 and at least one documented HAI between January 2020 and December 2022. Data were extracted from standardized reporting forms, and statistical analyses included tests (Fisher’s exact test, Mann–Whitney U), ROC curves, Kaplan–Meier survival analysis, and Cox proportional hazard regression. Results: The analysis revealed a mortality rate of 32.5%, significantly associated with advanced age, gastrointestinal surgery, and respiratory infections. *Clostridioides difficile* was the predominant pathogen (84.1%), and the threshold of ≥63.5 years demonstrated predictive value for mortality. In multivariate models, age greater than 63 years and gastrointestinal surgery were confirmed as independent predictors of death. Conclusions: The findings highlight the substantial impact of HAIs on the clinical progression of COVID-19 patients, underscoring the need for comprehensive systemic interventions, including enhanced prevention and control strategies, prudent antimicrobial therapy, and standardized epidemiological monitoring. Implementing these measures is crucial to mitigating HAIs’ effects and improving patient outcomes in similar situations.

## 1. Introduction

Healthcare-associated infections (HAIs) pose a significant global public health concern, directly affecting patient safety, the quality of healthcare services, and the sustainability of healthcare systems. According to World Health Organization data, millions of patients develop HAIs annually, leading to significant increases in morbidity, mortality, length of hospital stay, and healthcare costs [1,2]. The epidemiological impact is not evenly distributed, with prevalence considerably higher in low- and middle-income countries, where it can reach 15.5%, compared with approximately 7.1% in countries with advanced medical infrastructure [3,4]. The most common HAIs include ventilator-associated pneumonia, urinary tract infections, surgical site infections, septicemia, and gastrointestinal infections, particularly those caused by *Clostridioides difficile* [5,6].

The COVID-19 pandemic has increased patients’ vulnerability to HAIs, especially among those admitted to intensive care units. Overcrowding in medical facilities, extensive and often inappropriate use of antibiotics, reduced adherence to infection prevention and control measures, and the immunosuppressive effects of SARS-CoV-2 infection and adjuvant therapies have been important contributing factors [7,8]. Moreover, COVID-19 is now recognized as a systemic disease that affects multiple organ systems, including the lung, cardiovascular system [9], nervous system [10], and gastrointestinal tract [11], which may partially explain the prolonged hospitalization and increased susceptibility to healthcare-associated infections in these patients. Multicenter studies have reported an increase in the incidence of secondary bacterial and fungal infections, particularly ventilator-associated pneumonia and *C. difficile* infections, both of which are associated with severe clinical outcomes and increased mortality [12,13,14].

Epidemiological data on HAIs in Romania are inadequate and incomplete. Before the pandemic, the official reporting rate was below 1%, markedly lower than the European average of over 6%, suggesting persistent underreporting [15]. The pandemic exerted additional pressure on the healthcare system, leading to inconsistent protocols and limited resources, which contributed to incomplete MRSA documentation [2]. Recent studies have indicated a rise in *C. difficile* infection rates and a notable association between HAIs and mortality in COVID-19 patients; however, comprehensive epidemiological data at both the national and regional levels remain limited.

The examination of risk factors linked to mortality in patients with COVID-19 and healthcare-associated infections is crucial for enhancing prevention, triage, and treatment strategies, particularly in healthcare systems with constrained resources and a high prevalence of nosocomial infections [16]. In this context, local observational studies provide essential evidence to inform public health policies and improve clinical management. Based on these considerations, the present study aims to investigate clinical, epidemiological, and microbiological characteristics associated with mortality among patients hospitalized with COVID-19 and HAIs in a county located in Southeastern Romania.

## 2. Materials and Methods

### 2.1. Study Design and Population

This research was designed as a retrospective observational study, based on data reported to the Local Public Health Directorate by all healthcare facilities in the county. The reported infections originated exclusively from seven public hospitals, as private healthcare facilities in the county did not report any COVID-19 cases or related HAIs during the study period. Only patients with confirmed healthcare-associated infections recorded in the national surveillance system and reported by local hospitals during the study period were included in the analysis. The study period spanned from January 2020 to December 2022, coinciding with the critical stages of the COVID-19 pandemic, which enabled the assessment of HAIs within an epidemiological context marked by increased pressure on the healthcare system. A total of 295 hospitalized patients were included, selected based on the following eligibility criteria: confirmed SARS-CoV-2 infection by standard laboratory methods and clinical or microbiological documentation of at least 1 HAI. The types of infections investigated included *Clostridioides difficile* infections, respiratory infections, urinary tract infections, and other forms of HAIs reported during the same period.

Exclusion criteria targeted patients with incomplete data in the standardized reporting forms or with previous infectious episodes not attributed to the current hospitalization. This methodological approach enabled the creation of a representative cohort at the county level, providing a solid basis for analyzing clinical, epidemiological, and microbiological factors associated with mortality in patients with COVID-19 and HAIs.

For this study, healthcare-associated infections were defined according to the National Methodology for the Surveillance of Healthcare-Associated Infections [17], aligned with the case definitions established by the European Centre for Disease Prevention and Control (ECDC) [18]. The term multidrug-resistant infection (MDR infection) refers to infections caused by bacterial strains resistant to at least one antimicrobial agent in three or more antimicrobial classes, according to ECDC/CDC criteria [19,20]. To ensure terminological consistency, the term HAIs is used as the general category throughout this manuscript, while MDR infections are specified only when relevant to the microbiological or clinical analysis.

### 2.2. Variables Analyzed

The data used in the analysis were extracted from the standardized HAIs case form, an official document provided for in the National Methodology for the Surveillance of Healthcare-Associated Infections [17]. This form is a uniform tool for data collection at the national level, ensuring the comparability of results by including predefined categories of variables.

The information collected covered both the socio-demographic dimension and the clinical and microbiological profile of patients. Demographic variables included age, sex, and identification data associated with each case. The medical history encompassed pre-existing conditions, immune status, the presence or absence of immunosuppressive conditions, and specifics of prior surgical procedures, with a particular focus on gastrointestinal surgery. Regarding the spectrum of infections, the form allowed for the classification of the type of HAIs (*Clostridioides difficile* infections, respiratory infections, urinary tract infections, surgical site infections (SSIs) and other locations). The microbiological component included the isolation of pathogens and the identification of the bacterial or fungal species involved. Data regarding prior hospitalizations, antibiotic exposure, and utilized therapeutic regimens were documented, providing information pertinent to the analysis of antimicrobial resistance. The primary outcome variable was the clinical evolution of each patient, specifically survival or death.

This structure enabled the database to provide a comprehensive framework for evaluating the association between demographic, clinical, therapeutic, and microbiological factors and the mortality risk associated with MRD infections in COVID-19 patients.

### 2.3. Statistical Analysis

Data evaluation was performed using a set of statistical methods—Fisher’s exact tests, Mann–Whitney U tests, ROC curves, Kaplan–Meier survival analyses, and Cox proportional-hazard regression models—applied rigorously to ensure the robustness and validity of the results. Data processing was performed using IBM SPSS Statistics for Windows, version 25.0 (IBM Corp., Armonk, NY, USA). Graphical representations were generated using Microsoft Office Excel and Word 2024 (Microsoft Corp., Redmond, WA, USA).

Qualitative variables were described in terms of frequencies and percentages, with comparisons between groups performed using Fisher’s exact test. A detailed analysis of contingency tables was conducted using Z-tests with the Bonferroni correction. Quantitative variables were expressed as means and standard deviations or as medians with interquartile range (IQR), depending on the distribution. Normality was checked using the Shapiro–Wilk test, and comparisons of independent variables with nonparametric distributions were performed using the Mann–Whitney U test.

The relationship between age and mortality was evaluated through ROC curve analysis, which included the area under the curve (AUC), the 95% confidence interval, and the optimal threshold identified using the Youden index. Survival was assessed via the Kaplan–Meier method, with curve comparisons conducted using the Log-rank test. Independent predictors of mortality were determined through proportional-hazard Cox regression models, both multivariate and univariate, with results presented as hazard ratios (HR) and 95% confidence intervals. The criterion for statistical significance was established at α = 0.05.

### 2.4. Use of GenAI in Writing

As indicated in the submission form, we used Consensus to enhance the clarity and coherence of the text and to help organize the references according to the template format, and Grammarly to improve the accuracy and correctness of the English language.

In the revised version of our manuscript we used Chatgpt -4o to help organize the references according to the template format.

## 3. Results

### 3.1. Clinical and Demographic Profile of the Cohort

**Figure 1:**
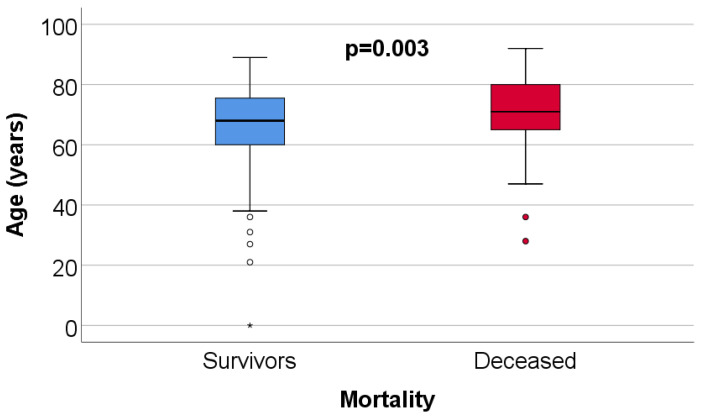
Box-plot figure illustrating the comparison of patients’ age according to mortality. Values representing circles in the figure are considered outliers, while values representing asterisk symbols are considered extreme outliers.

The study group included 295 patients diagnosed with COVID-19 and at least one healthcare-associated infection (HAIs). Of these, 199 (67.5%) survived and 96 (32.5%) died (Table 1). The median age of the cohort was 69 years (IQR: 61–77), but the comparative analysis showed significant differences between groups: deceased patients had a higher median age (71 years, IQR: 65–80) than survivors (68 years, IQR: 60–76; *p* = 0.003), a finding documented graphically in Figure 1. Demographically, the proportion of men was slightly higher (50.8%), and most patients came from urban areas (75.9%).

**Figure 2:**
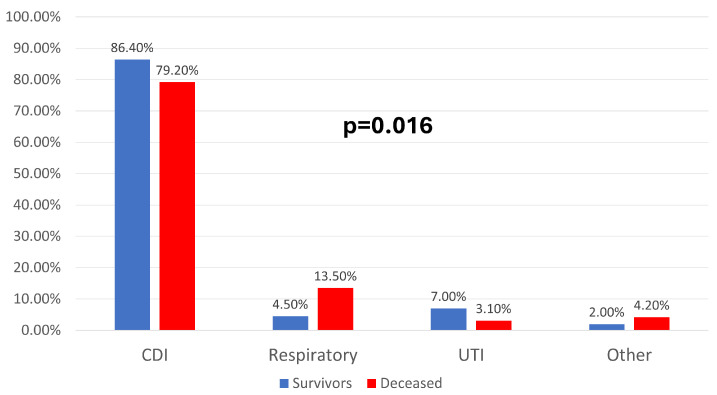
Distribution of patients according to mortality and type of HAIs (Healthcare-associated infections). CDI = *Clostridium difficile* infection, Respiratory = Respiratory healthcare-associated infections, UTI = Urinary tract infections, Other = Other healthcare-associated infections.

Etiological analysis revealed that *Clostridioides difficile* was the most common pathogen, accounting for 84.4% of cases, with statistically significant differences in clinical progression among groups (*p* = 0.016). Respiratory infections, although less common, were clearly correlated with mortality, being reported in 13.5% of deceased patients compared to 4.5% of survivors, a difference shown in Figure 2.

**Figure 3:**
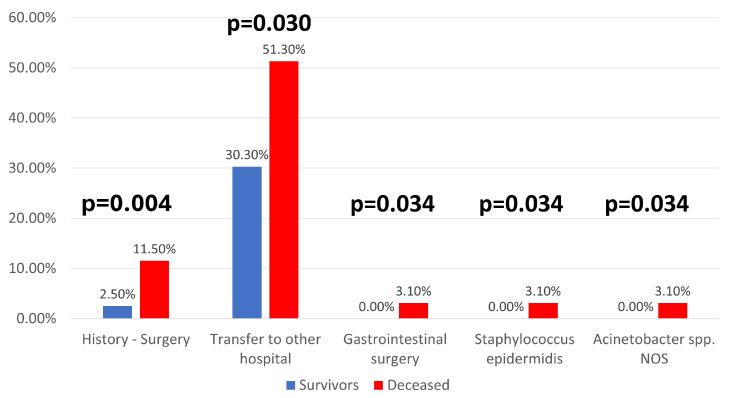
Distribution of patients according to mortality and medical history of surgery, gastrointestinal surgery, transfers to other hospitals, and isolation of *Staphylococcus epidermidis* or *Acinetobacter* spp. NOS = Not Otherwise Specified.

In terms of additional clinical factors, patients who required gastrointestinal surgery had a significantly higher death rate (3.1% vs. 0%; *p* = 0.034). At the same time, interhospital transfers were much more frequent in patients with fatal outcomes (51.3%) compared to survivors (30.3%; *p* = 0.030), as illustrated in Figure 3.

### 3.2. ROC (Receiver Operating Characteristic) Analysis

**Figure 4:**
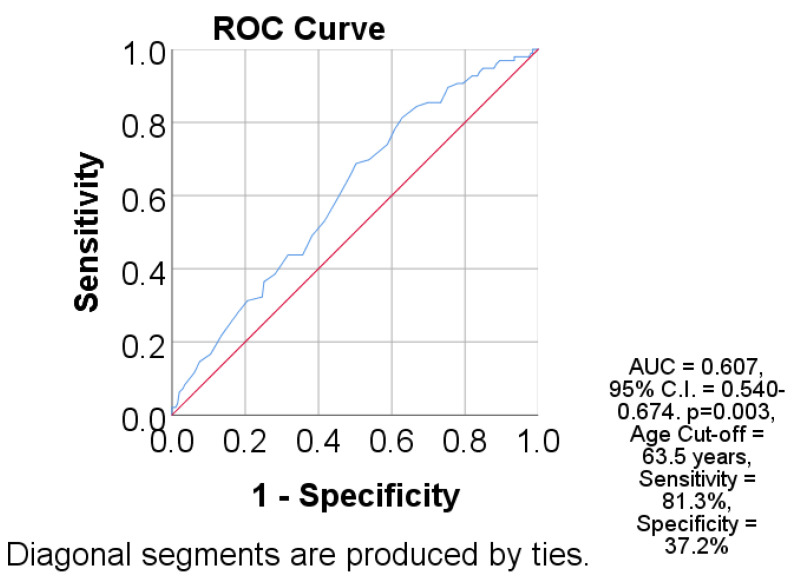
ROC (Receiver operating characteristic) curve analysis for the prediction of mortality using age. Age prediction evaluated in the blue line in the figure. AUC = Area under the curve, 95% C.I. = 95% Confidence interval for AUC. Cut-off obtained by selecting the value corresponding to the highest Youden index.

The Receiver Operating Characteristic (ROC) analysis was utilized to assess the predictive value of age concerning mortality, as shown in Figure 4. The ROC analysis indicated a low yet statistically significant discriminatory performance, evidenced by an AUC value of 0.607 (95% CI: 0.540–0.674; *p* = 0.003), thereby affirming the importance of age in risk stratification (Table 2). The identified optimal threshold was 63.5 years, associated with high sensitivity (81.3%) and moderate specificity (37.2%), emphasizing the importance of age as a significant factor in predictive models.

### 3.3. Kaplan–Meier Analysis

**Figure 5:**
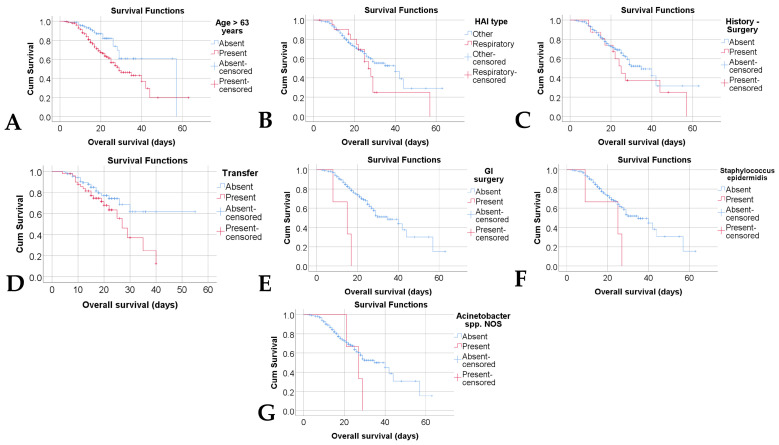
Kaplan–Meier comparison of overall survival according to age (**A**), HAI type (**B**), existence of surgery in the medical history (**C**), existence of transfers to other hospitals (**D**), existence of gastrointestinal surgery (**E**), existence of isolated Staphylococcus epidermidis (**F**) and existence of isolated *Acinetobacter* spp. NOS (**G**). Cum. survival = Cumulative survival, NOS = Not otherwise specified.

Kaplan–Meier curves were applied to estimate overall survival based on the clinical, epidemiological, and microbiological variables included in the analysis. The evaluation showed that the type of HAIs (*p* = 0.251)—Figure 5B, history of nonspecific surgical interventions (*p* = 0.276)—Figure 5C, and isolation of *Acinetobacter* spp. NOS strains (*p* = 0.211)—Figure 5F did not have a statistically significant impact on survival (Table 3).

In contrast, age proved to be a significant determinant: patients > 63 years of age had significantly lower overall survival (mean = 33.21 days, 95% CI: 28.21–38.21) compared to those ≤ 63 years (mean = 43.22 days, 95% CI: 37.33–49.11; *p* = 0.005), a difference illustrated in Figure 5A. A similar but even more pronounced effect was observed in patients undergoing gastrointestinal surgery, where the mean survival was significantly lower (13.33 days, 95% CI: 7.98–18.68) compared to patients without such procedures (36.14 days, 95% CI: 31.82–40.45; *p* < 0.001), a result reflected in Figure 5E.

Similarly, the isolation of *Staphylococcus epidermidis* was associated with reduced overall survival (20.33 days, 95% CI: 9.16–31.49) compared to patients in whom this agent was not identified (36.29 days, 95% CI: 31.93–40.65; *p* = 0.047), as shown in Figure 5F. A trend toward statistical significance was also observed in patients transferred between hospitals, who had slightly shorter survival times than those who were not transferred (*p* = 0.060), as shown in Figure 5D.

### 3.4. Cox Analysis

To identify independent factors associated with mortality, proportional-hazard Cox regression models were applied, developed in both univariate and multivariate variants. The results of the univariate analysis showed that both advanced age (*p* = 0.006) and gastrointestinal surgery (*p* = 0.001) were significant predictors of mortality. At the same time, the isolation of *Staphylococcus epidermidis* suggested only a marginal association (*p* = 0.061), which was not statistically confirmed (Table 4).

Integrating these variables into the multivariate model confirmed that age greater than 63 years and gastrointestinal surgery remain independent predictors of mortality. Specifically, patients aged >63 years had an almost twofold risk of death compared to younger patients (HR = 1.988; 95% CI: 1.188–3.327; *p* = 0.009), and patients undergoing gastrointestinal surgery had a more than fivefold increased risk of mortality (HR = 5.557; 95% CI: 1.731–17.845; *p* = 0.004) (Table 5).

## 4. Discussion

### 4.1. Types of Infections and Pathogens

Research published globally outlines an epidemiological profile of healthcare-associated infections during the COVID-19 pandemic, dominated by ventilator-associated pneumonia, vascular catheter-related bacteremia, and urinary tract infections, all of which are recognized as significant determinants of morbidity and mortality in intensive care units [21,22,23]. In addition, Gram-negative bacteria, such as *Acinetobacter baumannii, Pseudomonas* aeruginosa, and *Klebsiella pneumoniae*, have been consistently reported in multicenter studies in Europe and Asia, and are associated with a severe prognosis and increased rates of antimicrobial resistance [24]. Recent evidence has also demonstrated a significantly increased incidence of *Clostridioides difficile* infections during the pandemic, driven by antimicrobial pressure generated by the extensive use of broad-spectrum antibiotics, treatment-associated immunosuppression, and difficulties in uniformly implementing infection control strategies [25,26,27,28].

In this context, the data from our study reveal a distinct epidemiological feature: *C. difficile* was the primary etiological agent, identified in 84.1% of patients. This prevalence, which is significantly higher than that reported in other regions, can be attributed to the combination of intensive antibiotic use during the pandemic, the immunological vulnerability of hospitalized patients, and the challenges in implementing uniform HAIs prevention protocols. In contrast, respiratory and urinary tract infections had a low incidence, which differs from international trends and may reflect both the particularities of the population investigated and the limitations of local reporting.

The isolation of *Acinetobacter baumannii, Pseudomonas aeruginosa*, and *Klebsiella pneumoniae* corroborates trends documented in the literature, underscoring the pivotal role of these Gram-negative pathogens in HAIs and their substantial contribution to elevated mortality rates. These data indicate that *C. difficile* had a greater impact in the studied region than in other areas, underscoring the need to enhance epidemiological surveillance and antibiotic stewardship programs to reduce the risk of these infections.

### 4.2. Risk Factors

Identifying risk factors associated with mortality among patients with COVID-19 and healthcare-associated infections (HAIs) is a significant objective for optimizing prevention and clinical management strategies. Internationally published research has consistently shown that advanced age, immunosuppression, invasive procedures, and repeated hospitalizations significantly increase patients’ susceptibility to HAIs and negatively influence their prognosis [23,29]. Furthermore, factors such as invasive mechanical ventilation and high-dose corticosteroid administration have been identified as key determinants of mortality, with studies reporting that the association with HAIs can increase the risk of death by up to 8.5 times [30].

The results of the present study are consistent with these observations, confirming that advanced age and gastrointestinal surgery are independent predictors of mortality. Patients over the age of 63 had an almost twofold risk of death, reflecting the cumulative impact of biological frailty, comorbidities, and reduced immune reserves. Similarly, patients undergoing gastrointestinal surgery had a more than fivefold increase in mortality risk, which the complexity of the procedures can explain, the increased risk of postoperative complications, and the vulnerability associated with the critical conditions that necessitate such interventions.

These findings emphasize the need to promptly identify at-risk patients by incorporating age and surgical status into risk-stratification algorithms. Simultaneously, they advocate individualized treatment strategies, including enhanced surveillance of high-risk patients, judicious modification of corticosteroid and antibiotic regimens, and minimizing unwarranted exposure to invasive interventions. The execution of these techniques could diminish mortality rates among patients admitted with COVID-19 and HAIs.

### 4.3. Impact on Mortality and Survival

Numerous international studies have reported mortality rates associated with healthcare-associated infections in the context of COVID-19, ranging from 30% to 40%. This underscores the significant impact of these infections on the prognosis of hospitalized patients [31,32].

Multicenter analyses have shown that COVID-19 patients who develop HAIs have significantly higher mortality compared to those without such complications, and the study by Langlete et al. showed important differences between hospital-acquired and community-acquired infections, with a more severe course for the former. Interestingly, the same data suggest that SARS-CoV-2 vaccination has a significant protective effect, reducing the risk of death among these patients [33].

The results of our study confirm and extend these observations, showing a mortality rate of 32.5% in the analyzed cohort, which falls within the range reported in the literature. Kaplan–Meier analysis demonstrated significantly lower survival in patients aged >63 years and in those who required gastrointestinal surgery, highlighting the cumulative impact of biological frailty and the aggressiveness of invasive interventions on clinical outcome. In addition, the isolation of *Staphylococcus epidermidis* was associated with a significant reduction in survival; however, this relationship should be interpreted with caution, given the possibility of contamination in microbiological samples and the particular clinical context of patients with COVID-19.

The findings indicate that HAIs are a crucial factor influencing mortality and reduced survival in COVID-19 patients. This underscores the necessity of a comprehensive strategy that encompasses preventing secondary infections, meticulous risk stratification based on age and surgical status, and the implementation of tailored therapeutic approaches. Enhancing these methods may decrease mortality rates and enhance prognostic outcomes in comparable clinical situations.

### 4.4. National and International Context

Healthcare-associated infections pose a substantial public health challenge worldwide, characterized by high prevalence and significant effects on clinical outcomes and healthcare expenditures. Data from the ECDC indicates that from 2022 to 2023, around 4.3 million patients admitted to acute care hospitals in Europe acquired healthcare-associated infections, with nearly one-third classified as respiratory infections [12]. In the United States, CDC reports have shown comparable trends, with a significant increase in MD-associated HAIs during the pandemic, which directly contributed to worsening clinical outcomes and increased mortality [34].

In Romania, the official reporting rate of HAIs remained consistently below 1%, significantly lower than the European average of over 6%. This discrepancy reflects the structural shortcomings of the Romanian healthcare system in HAIs surveillance and reporting, deficiencies that were accentuated during the pandemic by resource pressure and the lack of uniform methodological frameworks [2,15].

This study’s results enhance the national epidemiological landscape by offering a comprehensive characterization of the clinical, microbiological, and demographic features of COVID-19 and HAIs patients in a representative county-level healthcare setting. The incorporation of these data within the international framework underscores the need to synchronize surveillance systems with European standards and to develop evidence-based public health policies that facilitate the implementation of effective infection prevention and control strategies.

### 4.5. Clinical Implications

This study’s results underscored significant clinical implications, emphasizing the necessity for enhanced integrated strategies to prevent and control healthcare-associated infections. Implementing hygiene and isolation protocols, along with applying epidemiological screening measures, is becoming essential to limit nosocomial transmission, especially in intensive care units and high-risk wards. The rational use of antibiotics is increasingly recognized as a strategic priority, necessitating the development of programs aimed at the judicious application of antimicrobial therapy. Such initiatives can mitigate the recurrence of infections and the emergence of bacterial resistance.

Although our dataset does not include information on SARS-CoV-2 vaccination, the recent literature suggests that vaccination may reduce disease severity and, indirectly, the risk of healthcare-associated infections, including *Clostridioides difficile* infections [33]. This highlights the importance of integrated preventive strategies that combine infection control measures and vaccination.

Moreover, minimizing unnecessary exposure to invasive medical devices and diligently monitoring patients undergoing complex surgical procedures are essential for reducing the risk of severe infectious complications. The incorporation of these measures into a structured institutional framework, underpinned by evidence-based public health policies and educational initiatives for medical personnel, is crucial for improving patient outcomes and strengthening the healthcare system’s resilience against future epidemiological challenges.

### 4.6. Strengths and Limitations of This Study

This study addresses a topic of significant clinical and epidemiological relevance: the impact of healthcare-associated infections on mortality in hospitalized patients with COVID-19. This retrospective observational study conducted in Romania provides valuable information in a context with limited surveillance and underreporting of HAIs.

The results of this study should be interpreted with certain methodological limitations in mind. The retrospective and monocentric nature of this research limits the generalizability of the conclusions, and the possibility of underreporting of healthcare-associated infections, coupled with the lack of complete microbiological information, may influence the accurate estimation of prevalence. Moreover, as this study relied on administrative data reported by hospitals to the Local Public Health Directorate, potential inconsistencies in diagnostic reporting and incomplete case validation may have introduced additional bias. Furthermore, the absence of detailed clinical variables—such as the severity of SARS-CoV-2 infection or the precise history of antibiotic exposure—introduces potential sources of confusion. These issues have already been highlighted by international research. Additionally, the ROC analysis (AUC = 0.607) indicated limited discriminatory ability, suggesting that the model’s predictive performance is modest and should be interpreted with caution rather than as a strong predictor. In this context, prospective multicenter studies are needed to validate the findings and enable more accurate identification of risk factors specific to the Romanian healthcare system [18,25].

## 5. Conclusions

This retrospective analysis highlights a substantial association between healthcare-associated infections (HAIs) and clinical outcomes among patients hospitalized with COVID-19 in a representative Romanian county. The observed mortality rate of 32.5% in the studied cohort reflects the relevance of HAIs in patient prognosis. Additionally, identifying advanced age and gastrointestinal surgery as independent risk factors underscores the need to develop targeted preventive and therapeutic strategies for at-risk populations.

The findings suggest that a multidimensional strategy encompassing enhanced infection prevention and control programs, prudent antibiotic use, and standardized epidemiological surveillance systems is important for reducing the incidence and severity of surgical site infections (SSIs). Implementing protocols for the meticulous monitoring of high-risk patients and restricting unnecessary exposure to invasive procedures may be associated with lower mortality rates.

This study provides pertinent local data in a context characterized by limited information on HAIs in Romania. The text presents compelling arguments for aligning the reporting and surveillance system with European standards. The findings align with the international literature, confirming the association of HAIs with increased mortality rates and underscoring the necessity for stringent infection control and prevention policies.

For future research, conducting prospective multicenter studies could validate these observations and provide a more accurate characterization of the risk factors and specific issues affecting the Romanian healthcare system while also facilitating the development of personalized interventions to improve patient outcomes.

## Figures and Tables

**Table 1:** Characteristics of the analyzed patients according to mortality.

Parameter (No., %)	Total	Survivors	Deceased	*p*
**N**	295 (100%)	199 (67.5%)	96 (32.5%)	-
Age (Median (IQR))	69 (61–77)	68 (60–76)	71 (65–80)	0.003 **
Gender (Male)	150 (50.8%)	106 (53.3%)	44 (45.8%)	0.264 *
Environment (Urban)	224 (75.9%)	151 (75.9%)	73 (76%)	1.000 *
***HAIs type***				
CDI	248 (84.1%)	172 (86.4%)	76 (79.2%)	0.016 *
Respiratory	22 (7.5%)	9 (4.5%)	13 (13.5%)	
UTI	17 (5.8%)	14 (7%)	3 (3.1%)	
Other	8 (2.7%)	4 (2%)	4 (4.2%)	
History-Surgery	16 (5.4%)	5 (2.5%)	11 (11.5%)	0.004 *
Isolated	262 (89.1%)	181 (91%)	81 (85.3%)	0.162 *
Infected contacts	56 (19.1%)	39 (19.7%)	17 (17.9%)	0.753 *
*HAIs classification*				
From the reporting hospital	180 (61%)	115 (57.8%)	65 (67.7%)	0.326 *
From another hospital	7 (2.4%)	6 (3%)	1 (1%)	
CDI	107 (36.3%)	77 (38.7%)	30 (31.3%)	
Chronic healthcare units	1 (0.3%)	1 (0.5%)	0 (0%)	
Immunodepression	34 (11.5%)	21 (10.6%)	13 (13.5%)	0.443 *
Transfer to another hospital	50 (36.2%)	30 (30.3%)	20 (51.3%)	0.030 *
Recurrence	10 (8.8%)	8 (9.8%)	2 (6.5%)	0.725 *
Complications	50 (16.9%)	28 (14.1%)	22 (22.9%)	0.069 *
Admission—Last year	84 (56.8%)	55 (51.9%)	29 (69%)	0.067 *
*Last admission*				
Less than 4 weeks ago	73 (90.1%)	47 (88.7%)	26 (92.9%)	0.843 *
4–12 weeks ago	3 (3.7%)	2 (3.8%)	1 (3.6%)	
>12 weeks ago	5 (6.2%)	4 (7.5%)	1 (3.6%)	
*Admission location*				
CDI reporting hospital	26 (31%)	19 (35.2%)	7 (23.3%)	0.328 *
Other hospital	58 (69%)	35 (64.8%)	23 (76.7%)	

* Fisher’s Exact Test, ** Mann–Whitney U Test. IQR = Interquartile range, HAI = Healthcare-associated infections, CDI = *Clostridium difficile* infection, UTI = Urinary tract infections. Data represents age median values with interquartile ranges or number of cases/column percentages for each nominal variable analyzed from the entire study group (Total), survivors or deceased patients.

**Table 2:** Treatment characteristics and isolated microorganisms of the analyzed patients according to mortality.

Parameter (No., %)	Total	Survivors	Deceased	*p*
**CDI—Antibiotic treatment**	214 (72.5%)	145 (72.9%)	69 (71.9%)	0.890 *
***No. Treatment sessions***				
One session	92 (43%)	68 (46.9%)	24 (34.8%)	0.106 *
>1 session	122 (57%)	77 (53.1%)	45 (65.2%)	
No. Antibiotics				
One	125 (58.4%)	90 (62.1%)	35 (50.7%)	0.138 *
Combination	89 (41.6%)	55 (37.9%)	34 (49.3%)	
Last 3 months—Treatment	73 (24.7%)	53 (26.6%)	20 (20.8%)	0.315 *
***No. Treatment sessions***				
One session	35 (47.9%)	24 (45.3%)	11 (55%)	0.600 *
>1 session	38 (52.1%)	29 (54.7%)	9 (45%)	
***No. Antibiotics***				
One	43 (58.9%)	33 (62.3%)	10 (50%)	0.426 *
Combination	30 (41.1%)	20 (37.7%)	10 (50%)	
Immunosuppressants	28 (9.5%)	18 (9%)	10 (10.4%)	0.678 *
Gastric antisecretory agents	113 (38.3%)	77 (38.7%)	36 (37.5%)	0.899 *
Gastrointestinal surgery	3 (1%)	0 (0%)	3 (3.1%)	0.034 *
Chemotherapy	3 (1%)	1 (0.5%)	2 (2.1%)	0.248 *
Contact with the CDI case	15 (5.1%)	11 (5.5%)	4 (4.2%)	0.780 *
A/B toxin	248 (84.1%)	172 (86.4%)	76 (79.2%)	0.127 *
PCR gene detection	5 (1.7%)	4 (2%)	1 (1%)	1.000 *
***Official HAIs-CDI source***				
Reporting hospital	239 (96%)	167 (96.5%)	72 (94.7%)	0.641 *
Other hospital	8 (3.2%)	4 (2.3%)	4 (5.3%)	
Outpatient unit	1 (0.4%)	1 (0.6%)	0 (0%)	
Chronic healthcare unit	1 (0.4%)	1 (0.6%)	0 (0%)	
***Identified microorganism***				
***Clostridium difficile***	249 (84.4%)	173 (86.9%)	76 (79.2%)	0.090 *
***Pseudomonas aeruginosa***	7 (2.4%)	7 (3.5%)	0 (0%)	0.100 *
***Escherichia coli***	6 (2%)	5 (2.5%)	1 (1%)	0.668 *
***Klebsiella*** **spp.** ***NOS***	2 (0.7%)	1 (0.5%)	1 (1%)	0.546 *
***Klebsiella oxytoca***	1 (0.3%)	1 (0.5%)	0 (0%)	1.000 *
***Klebsiella pneumonia***	6 (2%)	2 (1%)	4 (4.2%)	0.090 *
***Staphylococcus epidermidis***	3 (1%)	0 (0%)	3 (3.1%)	0.034 *
***Staphylococcus aureus***	3 (1%)	3 (1.5%)	0 (0%)	0.553 *
***Enterococcus*** **spp.** ***NOS***	5 (1.7%)	3 (1.5%)	2 (2.1%)	0.662 *
***Acinetobacter*** **spp.** ***NOS***	3 (1%)	0 (0%)	3 (3.1%)	0.034 *
***Acinetobacter baumannii***	9 (3.1%)	6 (3%)	3 (3.1%)	1.000 *
***Candida krusei***	2 (0.7%)	1 (0.5%)	1 (1%)	0.546 *
***Hafnia*** **spp.**	1 (0.3%)	1 (0.5%)	0 (0%)	1.000 *

* Fisher’s Exact Test, CDI = *Clostridium difficile* infection, HAI = Healthcare-associated infections, PCR = Polymerase Chain Reaction, NOS = Not Otherwise specified. Data represents number of cases/column percentages for each nominal variable analyzed from the entire study group (Total), survivors or deceased patients.

**Table 3:** ROC curve analysis for the prediction of mortality using age.

Parameter	AUC (95% C.I.)	Std. Error	*p*	Cut-Off	Se	Sp
Age	0.607 (0.540–0.674)	0.034	0.003	63.5	81.3	37.2

**Table 4:** Kaplan–Meier analyses for the overall survival comparison according to the investigated factors.

Age > 63 Years	Mean	95% C.I.	*p* *
Absent	43.22	37.33–49.11	0.005
Present	33.21	28.21–38.21	
*HAIs type*			
Other	37.33	32.14–42.52	0.251
Respiratory	31.38	23.13–39.62	
*History—Surgery*			
Absent	37.31	31.89–42.73	0.276
Present	31.77	22.25–41.30	
*Transfer*			
Absent	41.10	35.26–46.94	0.060
Present	26.44	22.22–30.66	
*GI surgery*			
Absent	36.14	31.82–40.45	<0.001
Present	13.33	7.98–18.68	
*Staphylococcus epidermidis*			
Absent	36.29	31.93–40.65	0.047
Present	20.33	9.16–31.49	
*Acinetobacter* spp. *NOS*			
Absent	36.24	31.95–40.72	0.211
Present	25.66	20.95–30.37	

* Log-rank test.

**Table 5:** Cox proportional-hazard regression, univariable and multivariable models, used in the prediction of mortality.

Parameter	Univariate		Multivariate	
	HR (95% CI)	*p*	HR (95% CI)	*p*
Age > 63 years	2.051 (1.228–3.426)	0.006	1.988 (1.188–3.327)	0.009
GI surgery	6.599 (2.063–21.103)	0.001	5.557 (1.731–17.845)	0.004
*Staphylococcus epidermidis*	3.014 (0.949–9.574)	0.061	-	-

## Data Availability

Data supporting the findings of this study are available from the corresponding author upon reasonable request.

## Abbreviations

The following abbreviations are used in this manuscript:

AUC	Area under the curve
CDC	Centers for Disease Control and Prevention
CDI	Clostridium difficile infection
CI	Confidence interval
ECDC	European Centre for Disease Prevention and Control
GI	Gastrointestinal
HAIs	Healthcare-Associated Infections
HR	Hazard ratios
IBM	International Business Machines
IBM Corp.	International Business Machines Corporation
IIC	Interval interquartile
IQR	Interquartile range
MDR infections	Multidrug-Resistant Infections
MRSA	Methicillin-Resistant Staphylococcus aureus
NOS	Not Otherwise Specified
NY	New York
*p*	*p*-value
PCR	Polymerase Chain Reaction
ROC	Receiver Operating Characteristic
se	Sensitivity
sp	Specificity
SPP	Species
SPSS	Statistical Package for the Social Sciences
SSI	Surgical Site Infection
USA	United States of America
UTI	Urinal tract infection
WHO	World Health Organization

## References

[1] World Health Organization (2011). Report on the Burden of Endemic Health Care-Associated Infection Worldwide.

[2] Allegranzi B., Bagheri Nejad S., Combescure C., Graafmans W., Attar H., Donaldson L., Pittet D. (2011). Burden of endemic health-care-associated infection in developing countries: Systematic review and meta-analysis. Lancet.

[3] Curtis S.J., Barnabas R., Cairns K.A., Cameron D., Coghlan B., Jones R., Joseph J., Kali A., Kep D., Klintworth G. (2024). Healthcare-associated infections and antimicrobial use at a major referral hospital in Papua New Guinea: A point prevalence survey. Lancet Reg. Health West Pac..

[4] Cassini A., Plachouras D., Eckmanns T., Abu Sin M., Blank H.P., Ducomble T., Haller S., Harder T., Klingeberg A., Sixtensson M. (2016). Burden of six healthcare-associated infections on European population health: Estimating incidence-based disability-adjusted life years through a population prevalence-based modelling study. PLoS Med..

[5] Magill S.S., O’Leary E., Janelle S.J., Thompson D.L., Dumyati G., Nadle J., Wilson L.E., Kainer M.A., Lynfield R., Greissman S. (2018). Changes in prevalence of health care-associated infections in U.S. hospitals. N. Engl. J. Med..

[6] Maillard J.Y., Bloomfield S.F., Courvalin P., Essack S.Y., Gandra S., Gerba C.P., Rubino J.R., Scott E.A. (2020). Reducing antibiotic prescribing and addressing the global problem of antibiotic resistance by targeted hygiene in the home and everyday life settings: A position paper. Am. J. Infect. Control.

[7] Rawson T.M., Moore L.S.P., Zhu N., Ranganathan N., Skolimowska K., Gilchrist M., Satta G., Cooke G., Holmes A. (2020). Bacterial and fungal coinfection in individuals with coronavirus: A rapid review to support COVID-19 antimicrobial prescribing. Clin. Infect. Dis..

[8] Garcia-Vidal C., Sanjuan G., Moreno-García E., Puerta-Alcalde P., Garcia-Pouton N., Chumbita M., Fernandez-Pittol M., Pitart C., Inciarte A., Bodro M. (2021). Incidence of co-infections and superinfections in hospitalized patients with COVID-19: A retrospective cohort study. Clin. Microbiol. Infect..

[9] Tudoran C., Tudoran M., Lazureanu V.E., Marinescu A.R., Cut T.G., Oancea C., Pescariu S.A., Pop G.N. (2021). Factors Influencing the Evolution of Pulmonary Hypertension in Previously Healthy Subjects Recovering from a SARS-CoV-2 Infection. J. Clin. Med..

[10] Molaverdi G., Kamal Z., Safavi M., Shafiee A., Mozhgani S.-H., Zarei Ghobadi M., Goudarzvand M. (2023). Neurological Complications after COVID-19: A Narrative Review. eNeurologicalSci..

[11] Groff A., Kavanaugh M., Ramgobin D., McClafferty B., Aggarwal C.S., Golamari R., Jain R. (2021). Gastrointestinal Manifestations of COVID-19: A Review of What We Know. Ochsner J..

[12] Lansbury L., Lim B., Baskaran V., Lim W.S. (2020). Co-infections in people with COVID-19: A systematic review and meta-analysis. J. Infect..

[13] Alqahtani A., Alamer E., Mir M., Alasmari A., Alshahrani M.M., Asiri M., Ahmad I., Alhazmi A., Algaissi A. (2022). Bacterial coinfections increase mortality of severely ill COVID-19 patients in Saudi Arabia. Int. J. Environ. Res. Public Health.

[14] Bentivegna E., Alessio G., Spuntarelli V., Luciani M., Santino I., Simmaco M., Martelletti P. (2021). Impact of COVID-19 prevention measures on risk of health care-associated *Clostridium difficile* infection. Am. J. Infect. Control.

[15] European Centre for Disease Prevention and Control (ECDC) (2023). Annual Epidemiological Report for 2018—Healthcare-Associated Infections Acquired in Intensive Care Units.

[16] Voinea C., Mocanu E., Opariuc-Dan C., Dantes E., Gache A.-C., Rugina S. (2025). Global lessons from COVID-19: Regional variations in the management of hospital-acquired infections during and post-pandemic. J. Clin. Med..

[17] National Center for Surveillance and Control of Communicable Diseases (CNSCBT) (2019). Annual Report on Healthcare-Associated Infections. https://www.cnscbt.ro.

[18] European Centre for Disease Prevention and Control (2024). Surveillance of Healthcare-Associated Infections and Prevention Indicators in European Acute Care Hospitals—HAI-Net Annual Report 2022.

[19] Centers for Disease Control and Prevention (2023). National Healthcare Safety Network (NHSN) Patient Safety Component Manual: Surgical Site Infection (SSI) Event.

[20] Magiorakos A.P., Srinivasan A., Carey R.B., Carmeli Y., Falagas M.E., Giske C.G., Harbarth S., Hindler J.F., Kahlmeter G., Olsson-Liljequist B.J. (2012). Multidrug-resistant, extensively drug-resistant and pandrug-resistant bacteria: An international expert proposal for interim standard definitions for acquired resistance. Clin. Microbiol. Infect..

[21] Ippolito M., Misseri G., Catalisano G., Marino C., Ingoglia G., Alessi M., Consiglio E., Gregoretti C., Giarratano A., Cortegiani A. (2021). Ventilator-Associated Pneumonia in Patients with COVID-19: A Systematic Review and Meta-Analysis. Antibiotics.

[22] Sciurti A., Baccolini V., Ceparano M., Isonne C., Migliara G., Iera J., Alessandri F., Ceccarelli G., Marzuillo C., Tellan G. (2024). Incidence and predictors of healthcare-associated infections in patients admitted to a temporary ICU during the COVID-19 pandemic Waves: A Two-Year (2021–2023) Retrospective Cohort Study in Rome, Italy. Antibiotics.

[23] Lewandowski K., Rosołowski M., Kaniewska M., Kucha P., Meler A., Wierzba W., Rydzewska G. (2021). Clostridioides difficile infection in coronavirus disease 2019 (COVID-19): An underestimated problem?. Pol. Arch. Intern. Med..

[24] Khavandegar A., Siami Z., Rasouli A., Nazemi P., Gull A. (2025). Healthcare-associated infections and mortality before and after COVID-19: A multicenter comparative analysis. Front. Public Health.

[25] Shabana H., Abdelwahed H. (2025). Impact of the COVID-19 pandemic on Clostridioides difficile infection rates in a tertiary hospital: A retrospective comparative study. Cureus.

[26] Granata G., Petrosillo N., Al Moghazi S., Caraffa E., Puro V., Tillotson G., Cataldo M.A. (2022). The burden of Clostridioides difficile infection in COVID-19 patients: A systematic review and meta-analysis. Anaerobe.

[27] Tsankof A., Protopapas A.A., Mantzana P., Protonotariou E., Skoura L., Protopapas N.D., Savopoulos C., Mimidis K. (2024). Clostridioides difficile infection in patients with and without COVID-19 during the pandemic: A retrospective cohort study from a tertiary referral hospital. Anaerobe.

[28] Nori P., Cowman K., Chen V., Bartash R., Szymczak W., Madaline T., Punjabi Katiyar C., Jain R., Aldrich M., Weston G. (2021). Bacterial and fungal coinfections in COVID-19 patients hospitalized during the New York city pandemic surge. Infect. Control Hosp. Epidemiol..

[29] Solís-Huerta F., Martinez-Guerra B.A., Roman-Montes C.M., Tamez-Torres K.M., Rajme-Lopez S., Ortíz-Conchi N., López-García N.I., Villalobos-Zapata G.Y., Rangel-Cordero A., Santiago-Cruz J. (2023). Risk factors associated with the development of hospital-acquired infections in hospitalized patients with severe COVID-19. Antibiotics.

[30] Trifi A., Sellaouti S., Mehdi A., Messaoud L., Seghir E., Tlili B., Abdellatif S. (2023). Healthcare-associated infections in critical COVID-19 patients: Impact on mortality and predictors. Acute Crit. Care.

[31] Zhou F., Yu T., Du R., Fan G., Liu Y., Liu Z., Xiang J., Wang Y., Song B., Gu X. (2020). Clinical course and risk factors for mortality of adult inpatients with COVID-19 in Wuhan, China: A retrospective cohort study. Lancet.

[32] Augello M., Castoldi R., Tavelli A., Nardo R., Sala V., Albertini L., Lundgren L.B., De Benedittis S., Borghi E., Viganò O. Incidence, risk factors and outcomes of healthcare-associated bacterial infections in COVID-19 patients receiving respiratory support: A retrospective cohort study. J. Infect. Public Health..

[33] Langlete P., Eriksen-Volle H.-M., Hessevik Paulsen T., Raastad R., Fagernes M., Bøås H., Himmels J. (2025). Healthcare-Associated COVID-19 Infections and Mortality. J. Hosp. Infect..

[34] Weiner-Lastinger L.M., Pattabiraman V., Konnor R.Y., Patel P.R., Wong E., Xu S.Y., Smith B., Edwards J.R., Dudeck M.A. (2022). The Impact of Coronavirus Disease 2019 (COVID-19) on Healthcare-Associated Infections in 2020: A Summary of Data Reported to the National Healthcare Safety Network. Infect. Control Hosp. Epidemiol..

